# 2-[(4-Chloro­phen­yl)sulfan­yl]-2-meth­oxy-1-phenyl­ethan-1-one: crystal structure and Hirshfeld surface analysis

**DOI:** 10.1107/S2056989018006072

**Published:** 2018-04-27

**Authors:** Ignez Caracelli, Julio Zukerman-Schpector, Henrique J. Traesel, Paulo R. Olivato, Mukesh M. Jotani, Edward R. T. Tiekink

**Affiliations:** a Departamento de Física, Universidade Federal de São Carlos, 13565-905 São Carlos, SP, Brazil; b Departamento de Química, Universidade Federal de São Carlos, 13565-905 São Carlos, SP, Brazil; c Instituto de Química, Universidade de São Paulo, 05508-000 São Paulo, SP, Brazil; d Department of Physics, Bhavan’s Sheth R. A. College of Science, Ahmedabad, Gujarat 380001, India; e Research Centre for Crystalline Materials, School of Science and Technology, Sunway University, 47500 Bandar Sunway, Selangor Darul Ehsan, Malaysia

**Keywords:** crystal structure, sulfan­yl, phenyl­ethanone, Hirshfeld surface analysis, NCI plots

## Abstract

The title compound, C_15_H_13_ClO_2_S, comprises (4-chloro­phen­yl)sulfanyl, benzaldehyde and meth­oxy residues linked at a chiral methine-C atom (the crystal is racemic). A twist in the methine-C—C(carbon­yl) bond [O—C—C—O torsion angle = 19.3 (7)°] leads to a dihedral angle of 22.2 (5)° between the benzaldehyde and methine+meth­oxy residues. The chloro­benzene ring is folded to lie over the O atoms, with the dihedral angle between the benzene rings being 42.9 (2)°. In the crystal, the carbonyl-O atom accepts two C—H⋯O inter­actions with methyl- and methine-C—H atoms being the donors. The result is an helical supra­molecular chain aligned along the *c* axis; chains pack with no directional inter­actions between them. An analysis of the Hirshfeld surface points to the important contributions of weak H⋯H and C⋯C contacts to the mol­ecular packing.

## Abstract 2

A chiral methine-C atom connects the (4-chloro­phen­yl)sulfanyl, benzaldehyde and meth­oxy residues in the racemic title compound. Supra­molecular helical chains are formed in the crystal, being sustained by methyl- and methine-C—H⋯O(carbon­yl) inter­actions.

## Chemical context   

As part of our ongoing studies on the conformational and electronic characteristics of some β-thio­carbonyl, β-bis-thio­carbonyl and β-thio-β-oxacarbonyl compounds, *e.g. N*,*N*-diethyl-2-[(4′-substituted)phenyl­thio]­acetamides (Vinhato *et al.*, 2013 ▸), 1-methyl-3-phenyl­sulfonyl-2-piperidones (Zuker­man-Schpector *et al.*, 2008 ▸), 3,3-bis­[(4′-substituted) phenyl­sulfan­yl]-1-methyl-2-piperidones (Olivato *et al.*, 2013 ▸), 2-alkyl­thio-2-alkyl­sulfinyl-aceto­phenones and 2-alkyl­thio-2-phenyl­sulfonyl-aceto­phenones, 2-alkyl­sulfinyl-2-alkyl­sulfonyl-aceto­phenones (Distefano *et al.*, 1996 ▸), 2-meth­oxy-2-[(4′-substituted) phenyl­sulfan­yl]-aceto­phenones (Zukerman-Schpector *et al.*, 2015 ▸; Caracelli *et al.*, 2015 ▸) and 2-meth­oxy-2-(phenyl­selan­yl)-(4′-substituted)aceto­phenones (Traesel *et al.*, 2018 ▸), utilizing infrared spectroscopy, computational chemistry and X-ray diffraction methods, the title compound (I) was synthesized and characterized. The primary motivation behind this work is the search for selenium/sulfur-containing compounds with anti-inflammatory activity that could be selective COX-2 inhibitors (Cerqueira *et al.*, 2015 ▸, 2017 ▸). Mol­ecular docking studies have also been conducted in order to understand the mechanism of inhibition (Baptistini, 2015 ▸). Herein, the crystal and mol­ecular structures of (I) are described along with an analysis of the calculated Hirshfeld surfaces and non-covalent inter­action plots for selected inter­actions.
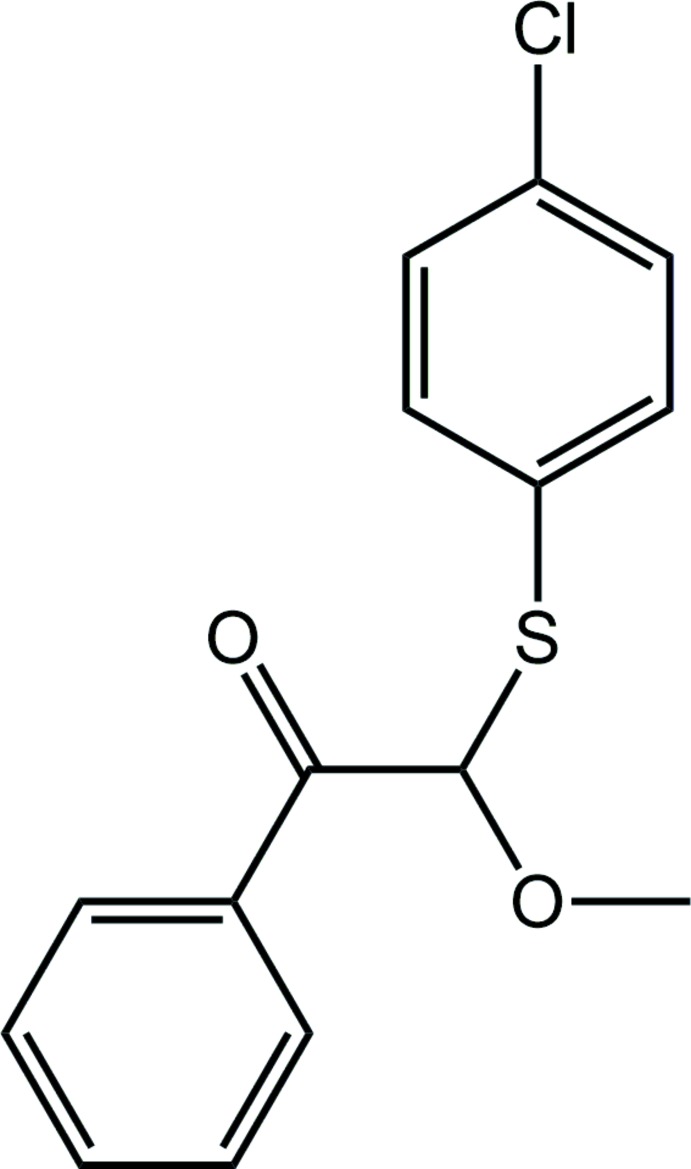


## Structural commentary   

**Figure 1:**
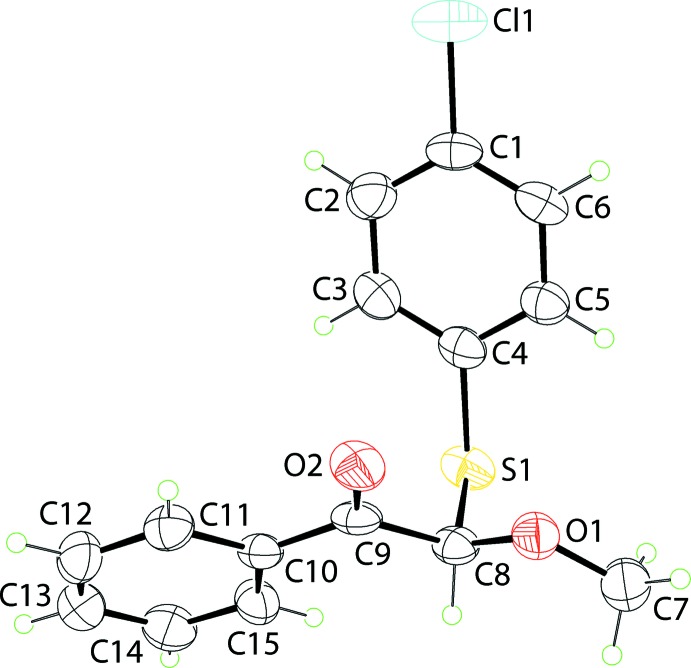
The mol­ecular structure of (I), showing the atom-labelling scheme and displacement ellipsoids at the 35% probability level.

The mol­ecular structure of (I) sees (4-chloro­phen­yl)sulfanyl, phenyl­ethanone and meth­oxy groups linked at the chiral methine-C8 atom, Fig. 1 ▸. In the arbitrarily chosen asymmetric mol­ecule, C8 has an *R* configuration, but crystal symmetry generates a racemic mixture. The base of the mol­ecule is defined by the phenyl­ethanone [r.m.s. deviation of the eight non-hydrogen atoms = 0.0134 Å] and meth­oxy groups. These residues are not co-planar, with the dihedral angle between the two planes being 22.2 (5)° owing to the twist about the C8—C9 bond as seen in the value of the O1—C8—C9—O2 torsion angle of 19.3 (7)°. The 4-chloro­phenyl group is orientated so that the ring lies over the oxygen atoms with the dihedral angle between the benzene rings being 42.9 (2)°.

## Supra­molecular features   

**Figure 2:**
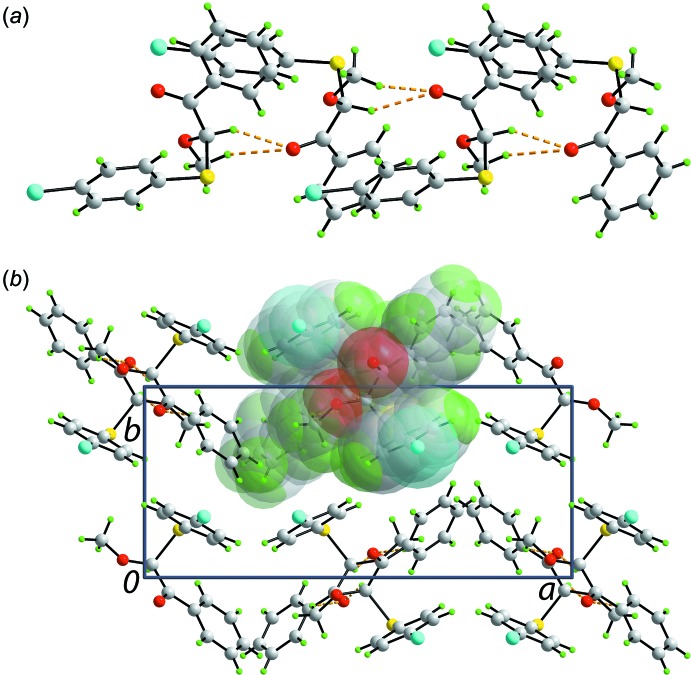
Mol­ecular packing in (I): (*a*) view of the supra­molecular chain parallel to the *c* axis and (*b*) view of the unit-cell contents shown in projection down the *b* axis; one chain is highlighted in space-filling mode. The C—H⋯O contacts are shown as orange dashed lines.

The mol­ecular packing of (I) features C—H⋯O inter­actions where the donors are methyl-C7 and methine-C8 H atoms, and the acceptor is the carbonyl-O2 atom, Table 1 ▸. These inter­actions combine to sustain a supra­molecular chain along [001] with an helical topology as it is propagated by 2_1_ symmetry, Fig. 2 ▸ *a*. Chains assemble into the three-dimensional architecture without directional inter­actions between them, Fig. 2 ▸ *b*.

## Hirshfeld surface analysis   

**Figure 3:**
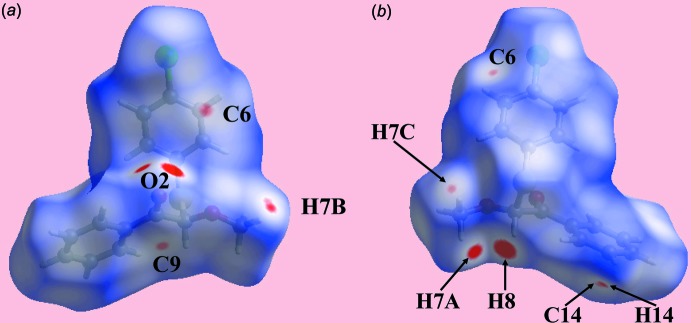
Two views of the Hirshfeld surface for (I) mapped over *d*_norm_ in the range −0.073 to +1.389 au.

**Figure 4:**
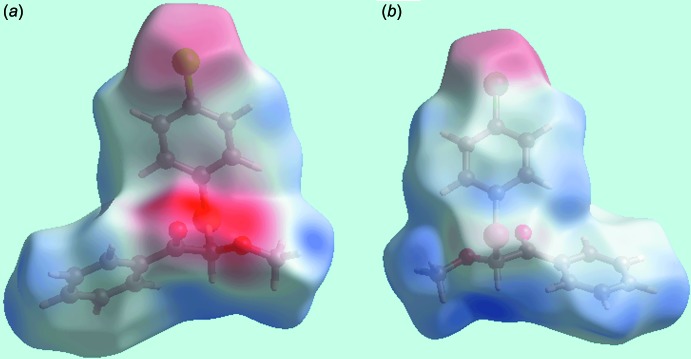
Two views of the Hirshfeld surfaces mapped over the electrostatic potential in the range −0.073 to + 0.056 au. The red and blue regions represent negative and positive electrostatic potentials, respectively.

**Figure 5:**
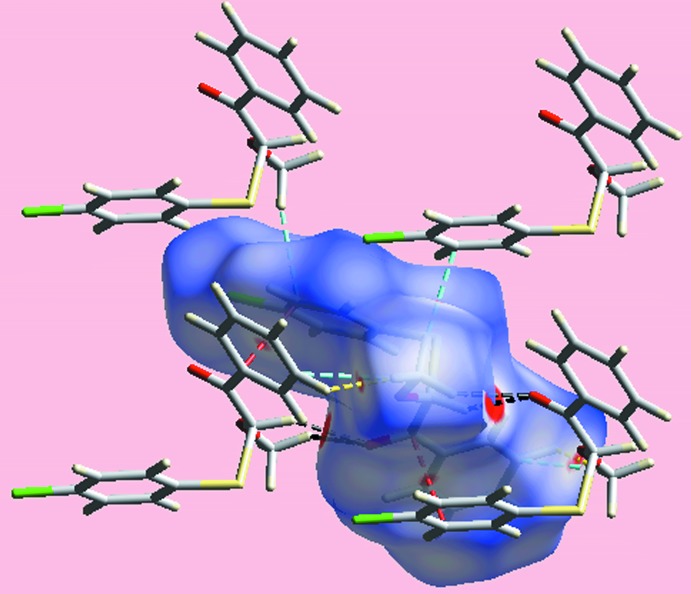
A view of the Hirshfeld surface mapped over *d*_norm_ in the range −0.073 to +1.389 au highlighting inter­molecular C—H⋯O, C⋯C, H⋯H and C⋯H/H⋯C contacts by black, red, yellow and sky-blue dashed lines, respectively.

The Hirshfeld surface calculations for (I) were performed as per a recent study (Zukerman-Schpector *et al.*, 2017 ▸) and serve to provide additional information on the mol­ecular packing, in particular the weaker inter­actions between mol­ecules. In addition to bright-red spots near the methyl-H7*A* and methine-H8 atoms, a pair near the carbonyl-O2 atom arise as a result of the C—H⋯O inter­actions leading to the supra­molecular chain discussed above, Table 1 ▸. The presence of diminutive and faint-red spots on the Hirshfeld surfaces illustrated in Fig. 3 ▸ indicate the influence of short inter­atomic contacts on the mol­ecular packing in the crystal, Table 2 ▸. Thus, the C⋯C and C⋯H/H⋯C contacts involving chloro­benzene-C6, carbonyl-C9 and methyl-H7*C* atoms are viewed as the pair of diminutive and faint-red spots near these atoms in Fig. 3 ▸, whereas similar features near the methyl-H7*B*, phenyl-C14 and -H14 atoms represent H7*B*⋯H14 and C⋯H/H⋯C contacts. Views of the Hirshfeld surfaces mapped over electrostatic potential are shown in Fig. 4 ▸ and also indicate the donors and acceptors of the C—H⋯O inter­actions through the appearance of intense-blue and -red regions around the participating atoms. Fig. 5 ▸ illustrates the environment around a reference mol­ecule within the *d*_norm_-mapped Hirshfeld surface and highlight the inter­molecular C—H⋯O inter­actions and short inter­atomic H⋯H, C⋯H/H⋯C and C⋯C contacts.

**Figure 6:**
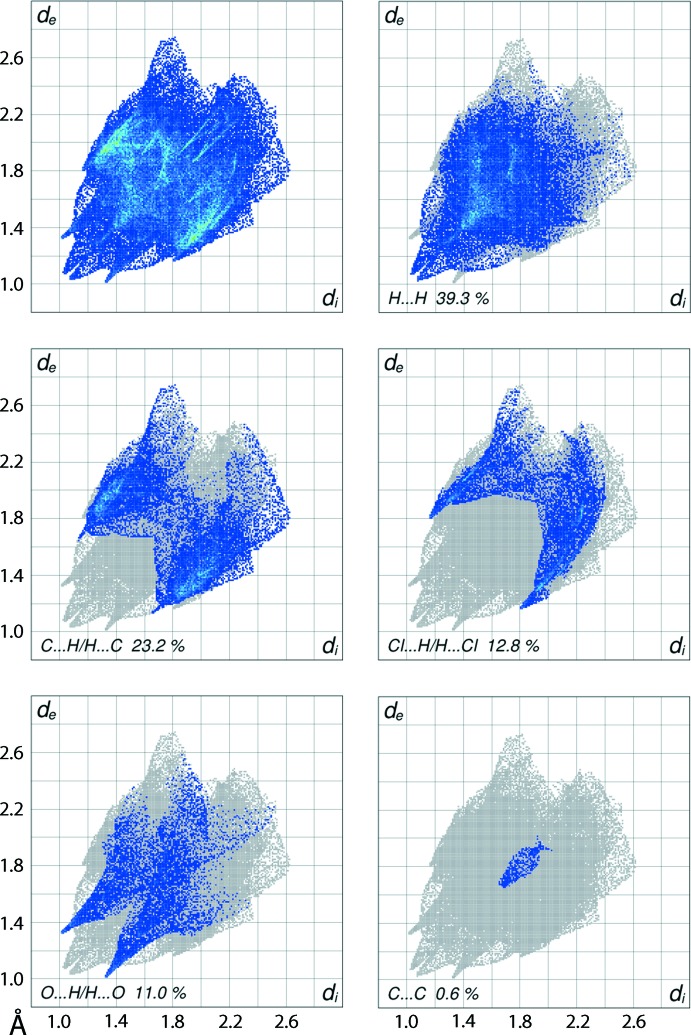
The full two-dimensional fingerprint plot for (I) and those delineated into H⋯H, C⋯H/H⋯C, Cl⋯H/H⋯Cl, O⋯H/H⋯O and C⋯C contacts.

The non-symmetric mol­ecular geometry in (I) results in an asymmetric distribution of points in its overall two-dimensional fingerprint plot shown in Fig. 6 ▸ and also in those delin­eated into H⋯H, C⋯H/H⋯C, Cl⋯H/H⋯Cl, O⋯H/H⋯O and C⋯C contacts (McKinnon *et al.*, 2007 ▸), also illus­trated in Fig. 6 ▸. The major percentage contributions to the Hirshfeld surface are from (in descending order) H⋯H, C⋯H/H⋯C, Cl⋯H/H⋯Cl, O⋯H/H⋯O and S⋯H/H⋯S contacts along with a small, *i.e*. 0.6%, contribution from C⋯C contacts as summarized in Table 3 ▸. These inter­actions result in distinctive features in their respective delineated fingerprint plots. The short inter­atomic H⋯H and C⋯H/H⋯C contacts are characterized as a pair of beak-shape tips at *d*_e_ + *d*_i_ ∼ 2.1 Å and the pair of parabolic distributions of points at around *d*_e_ + *d*_i_ < 2.8 Å in their respective delineated fingerprint plots. The short inter­atomic C⋯H/H⋯C contacts in the crystal, Table 2 ▸, appear as a pair of thin tips at *d*_e_ + *d*_i_ ∼ 2.7 Å attached to the aforementioned parabolic distribution. The inter­atomic Cl⋯H/H⋯Cl contacts, making the next most significant contribution to the Hirshfeld surface, *i.e*. 12.8%, are at van der Waals separations. The C—H⋯O contacts, involving the carbonyl-O2 with methyl-C7 H and methine-C8 H atoms, Table 1 ▸, are evident as a pair of spikes with tips at *d*_e_ + *d*_i_ ∼ 2.3 Å. The vase-shaped distribution of points beginning at *d*_e_ + *d*_i_ ∼ 3.3 Å in the fingerprint plot delineated into C⋯C contacts results from the contacts highlighted in Fig. 5 ▸ and Table 2 ▸. The small contribution from other remaining inter­atomic contacts summarized in Table 3 ▸ have a negligible influence upon the mol­ecular packing.

## Non-covalent inter­action plots   

**Figure 7:**
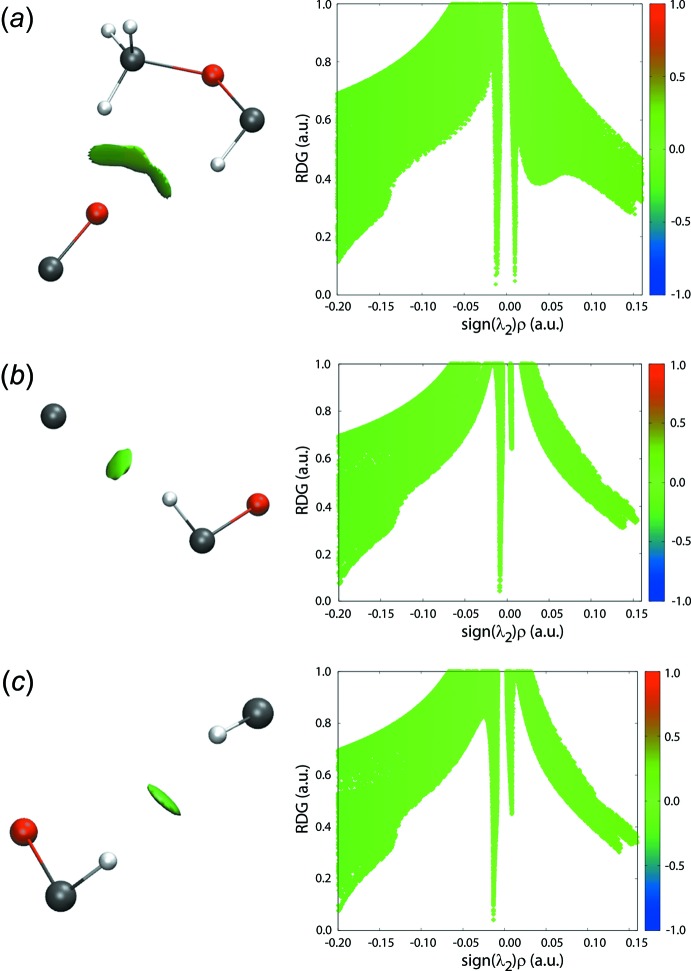
Non-covalent inter­action plots for inter­molecular inter­actions between (*a*) methyl-C7- and methine-C—H atoms, and the carbonyl-O2 atom, (*b*) chloro­benzene-C6 and methyl-H7*C* atoms and (*c*) methyl-H7*B* and phenyl-H14 atoms.

Non-covalent inter­action plots are a convenient means by which the nature of a specified inter­molecular inter­action may be assessed in terms of it being attractive or otherwise (Johnson *et al.*, 2010 ▸; Contreras-García *et al.*, 2011 ▸). If a specified inter­action is attractive, the isosurface will be blue in appearance whereas a repulsive inter­action will result in a red isosurface. On the other hand, a weakly attractive inter­action will appear green. The isosurfaces for the inter­actions between the methyl-C7 and methine-C H atoms and the carbonyl-O2 atom are shown in Fig. 7 ▸ *a*, clearly indicating their weakly attractive nature. Similarly, the inter­actions between the chloro­benzene-C6 and methyl-H7*C* atoms, Fig. 7 ▸ *b*, and between the methyl-H7*B* and phenyl-H14 atoms, Fig. 7 ▸ *c*, are weakly attractive.

## Database survey   

**Figure 8:**
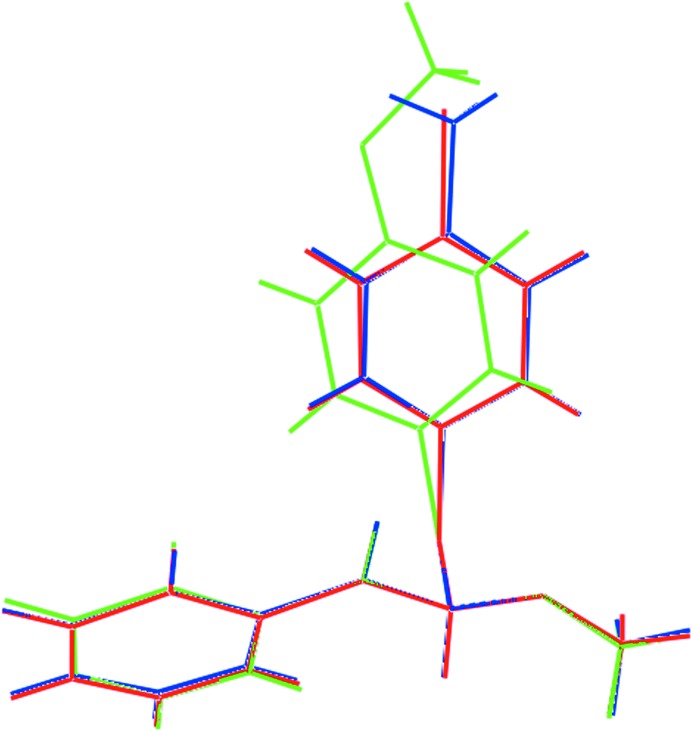
Overlay diagram of (*a*) (I), red image, (*b*) (II), green and (*c*) (III), blue.

There are two closely related literature precedents for (I), namely the S-bound 4-meth­oxy­benzene [(II); Caracelli *et al.*, 2015 ▸] and 4-tolyl [(III); Zukerman-Schpector *et al.*, 2015 ▸] derivatives. The three compounds crystallize in the same *Pca*2_1_ space group and present similar unit-cell dimensions. An overlay diagram for (I)–(III) is shown in Fig. 8 ▸ from which it can be noted there is a high degree of concordance for (I) and (III). The mol­ecule in (II) is coincident with (I) and (III) except for the relative disposition of the S-bound meth­oxy­benzene ring. This difference arises as a result of a twist about the C8—S1 bond as seen in the C4—S1—C8—C9 torsion angles of 57.3 (5), 46.6 (3) and 57.9 (3)° for (I)–(III), respectively. Despite this difference, the angles between the S-bound benzene rings and the phenyl rings in (I)–(III) are relatively constant at 42.9 (2), 40.11 (16) and 44.03 (16)°, respectively.

## Synthesis and crystallization   

The 4′-chloro­phenyl di­sulfide precursor was prepared as previously described (Ali & McDermott, 2002 ▸) through the oxidation of 4′-chloro­thio­phenol by bromine. A solution of 2-meth­oxy aceto­phenone (0.70 ml, 5.08 mmol, Sigma–Aldrich) in THF (15 ml), was added dropwise to a cooled (195 K) solution of diiso­propyl­amine (0.78 ml, 5.59 mmol) and *n*-butyl­lithium (3.76 ml, 5.08 mmol) in THF (25 ml). After 30 min., a solution of 4′-chloro­phenyl di­sulfide (1.61 g, 5.08 mmol) with hexa­methyl­phospho­ramide (HMPA) (0.90 ml, *ca* 5.08 mmol) dissolved in THF (15 ml) was added dropwise to the enolate solution (Zoretic & Soja, 1976 ▸). After stirring for 3 h, water (50 ml) was added at room temperature and extraction with diethyl ether was performed. The organic layer was then treated with a saturated solution of ammonium chloride until neutral pH and dried over anhydrous magnesium sulfate. A brown oil was obtained after evaporation of solvent. Purification through flash chromatography with *n*-hexane was used in order to remove the non-polar reactant (di­sulfide), then with dry acetone to give a mixture of both aceto­phenones (product and reactant). Crystallization was performed by vapour diffusion of *n*-hexane into a chloro­form solution held at 283 K to give pure product (0.4 g, yield = 60%). Irregular colourless crystals for X-ray diffraction of (I) were obtained by the same pathway. M.p. 358.2–358.8 K. ^1^H NMR (CDCl_3_, 500 MHz, δ ppm): 3.67 (*s*, 3H), 5.86 (*s*, 1H), 7.24–7.29 (*m*, 4H), 7.44–7.47 (*m*, 2H), 7.57–7.60 (*m*, 1H), 7.93–7.95 (m, 2H). ^13^C NMR (CDCl_3_, 125 MHz, δ p.p.m.): 190.20, 135.60, 135.25, 134.23, 133.55, 129.22, 128.84, 128.59, 89.37, 56.13. Microanalysis calculated for C_15_H_13_ClO_2_S (%): C 61.53, H 4.48. Found (%): C 61.47, H 4.55. High-resolution MS [*M* ^+^, *M* ^2+^] calculated: 292.0325, 294.0295; found: 292.0324, 294.0296.

## Refinement details   

Crystal data, data collection and structure refinement details are summarized in Table 4 ▸. The carbon-bound H atoms were placed in calculated positions (C—H = 0.93–0.98 Å) and were included in the refinement in the riding-model approximation, with *U*_iso_(H) set to 1.2–1.5*U*_eq_(C).

## Supplementary Material

Crystal structure: contains datablock(s) I, global. DOI: 10.1107/S2056989018006072/hb7746sup1.cif

Structure factors: contains datablock(s) I. DOI: 10.1107/S2056989018006072/hb7746Isup2.hkl

Supporting information file. DOI: 10.1107/S2056989018006072/hb7746Isup3.cml

CCDC reference: 1838590

Additional supporting information:  crystallographic information; 3D view; checkCIF report

## Figures and Tables

**Table 1:** Hydrogen-bond geometry (Å, °)

*D*—H⋯*A*	*D*—H	H⋯*A*	*D*⋯*A*	*D*—H⋯*A*
C7—H7*A*⋯O2^i^	0.96	2.53	3.297 (9)	137
C8—H8⋯O2^i^	0.98	2.42	3.305 (8)	150

Symmetry code: (i) 
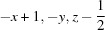
.

**Table 2:** Summary of short inter­atomic contacts (Å) in (I)

Contact	Distance	Symmetry operation
H7*B*⋯H14	2.10	1 − *x*, − *y*, 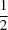 + *z*
H7*B*⋯C14	2.76	1 − *x*, − *y*, 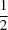 + *z*
H7*C*⋯C6	2.73	1 − *x*, 1 − *y*, 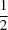 + *z*
C6⋯C9	3.33	1 − *x*, − *y*, 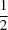 + *z*

**Table 3:** Percentage contributions of inter­atomic contacts to the Hirshfeld surface for (I)

Contact	Percentage contribution
H⋯H	39.3
C⋯H/H⋯C	23.2
Cl⋯H/H⋯Cl	12.8
O⋯H/H⋯O	11.0
S⋯H/H⋯S	4.4
Cl⋯S/S⋯Cl	2.1
Cl⋯O/O⋯Cl	2.1
C⋯O/O⋯C	1.5
C⋯Cl/Cl⋯C	1.5
C⋯S/S⋯C	1.2
C⋯C	0.6

**Table 4:** Experimental details

Crystal data	
Chemical formula	C_15_H_13_ClO_2_S
*M*_r_	292.76
Crystal system, space group	Orthorhombic, *P* *c* *a*2_1_
Temperature (K)	293
*a*, *b*, *c* (Å)	17.964 (3), 8.0234 (15), 9.7761 (19)
*V* (Å^3^)	1409.0 (5)
*Z*	4
Radiation type	Mo *K*α
μ (mm^−1^)	0.41
Crystal size (mm)	0.42 × 0.21 × 0.12

Data collection	
Diffractometer	Bruker APEXII CCD
Absorption correction	Multi-scan (*SADABS*; Sheldrick, 1996 ▸)
*T*_min_, *T*_max_	0.365, 0.745
No. of measured, independent and observed [*I* > 2σ(*I*)] reflections	5010, 2081, 1505
*R*_int_	0.049
(sin θ/λ)_max_ (Å^−1^)	0.594

Refinement	
*R*[*F* ^2^ > 2σ(*F* ^2^)], *wR*(*F* ^2^), *S*	0.048, 0.116, 1.04
No. of reflections	2081
No. of parameters	173
No. of restraints	1
H-atom treatment	H-atom parameters constrained
Δρ_max_, Δρ_min_ (e Å^−3^)	0.28, −0.18
Absolute structure	Flack *x* determined using 465 quotients [(*I* ^+^)−(*I* ^−^)]/[(*I* ^+^)+(*I* ^−^)] (Parsons *et al.*, 2013 ▸)
Absolute structure parameter	0.06 (9)

Computer programs: *APEX2* and *SAINT* (Bruker, 2009 ▸), *SIR2014* (Burla *et al.*, 2015 ▸), *SHELXL2014* (Sheldrick, 2015 ▸), *ORTEP-3 for Windows* (Farrugia, 2012 ▸), *DIAMOND* (Brandenburg, 2006 ▸), *MarvinSketch* (ChemAxon, 2010 ▸) and *publCIF* (Westrip, 2010 ▸).

## supplementary crystallographic information

### Crystal data

**Table:**

C_15_H_13_ClO_2_S	*D*_x_ = 1.380 Mg m^−^^3^
*M**_r_* = 292.76	Mo *K*α radiation, λ = 0.71073 Å
Orthorhombic, *P**c**a*2_1_	Cell parameters from 1839 reflections
*a* = 17.964 (3) Å	θ = 2.5–23.7°
*b* = 8.0234 (15) Å	µ = 0.41 mm^−^^1^
*c* = 9.7761 (19) Å	*T* = 293 K
*V* = 1409.0 (5) Å^3^	Irregular, colourless
*Z* = 4	0.42 × 0.21 × 0.12 mm
*F*(000) = 608	

### Data collection

**Table:**

Bruker APEXII CCD diffractometer	1505 reflections with *I* > 2σ(*I*)
φ and ω scans	*R*_int_ = 0.049
Absorption correction: multi-scan (SADABS; Sheldrick, 1996)	θ_max_ = 25.0°, θ_min_ = 2.3°
*T*_min_ = 0.365, *T*_max_ = 0.745	*h* = −20→21
5010 measured reflections	*k* = −7→9
2081 independent reflections	*l* = −11→8

### Refinement

**Table:**

Refinement on *F*^2^	Hydrogen site location: inferred from neighbouring sites
Least-squares matrix: full	H-atom parameters constrained
*R*[*F*^2^ > 2σ(*F*^2^)] = 0.048	*w* = 1/[σ^2^(*F*_o_^2^) + (0.043*P*)^2^ + 0.3264*P*] where *P* = (*F*_o_^2^ + 2*F*_c_^2^)/3
*wR*(*F*^2^) = 0.116	(Δ/σ)_max_ < 0.001
*S* = 1.04	Δρ_max_ = 0.28 e Å^−^^3^
2081 reflections	Δρ_min_ = −0.18 e Å^−^^3^
173 parameters	Absolute structure: Flack *x* determined using 465 quotients [(*I*^+^)-(*I*^-^)]/[(*I*^+^)+(*I*^-^)] (Parsons *et al.*, 2013)
1 restraint	Absolute structure parameter: 0.06 (9)

### Special details

**Table:**

Geometry. All esds (except the esd in the dihedral angle between two l.s. planes) are estimated using the full covariance matrix. The cell esds are taken into account individually in the estimation of esds in distances, angles and torsion angles; correlations between esds in cell parameters are only used when they are defined by crystal symmetry. An approximate (isotropic) treatment of cell esds is used for estimating esds involving l.s. planes.

### Fractional atomic coordinates and isotropic or equivalent isotropic displacement parameters (Å^2^)

**Table:**

	*x*	*y*	*z*	*U*_iso_*/*U*_eq_	
Cl1	0.36121 (11)	0.3177 (3)	1.0687 (2)	0.0916 (7)	
S1	0.41934 (10)	0.2492 (2)	0.4390 (2)	0.0646 (5)	
O1	0.5511 (2)	0.0919 (5)	0.4909 (4)	0.0616 (11)	
O2	0.4654 (2)	−0.1253 (5)	0.6070 (4)	0.0685 (12)	
C1	0.3786 (4)	0.2943 (8)	0.8946 (7)	0.0571 (17)	
C2	0.3254 (4)	0.2193 (8)	0.8147 (9)	0.069 (2)	
H2	0.2812	0.1806	0.8527	0.083*	
C3	0.3396 (4)	0.2028 (8)	0.6744 (8)	0.0644 (18)	
H3	0.3048	0.1506	0.6186	0.077*	
C4	0.4045 (3)	0.2632 (7)	0.6186 (7)	0.0529 (15)	
C5	0.4565 (4)	0.3385 (7)	0.7019 (7)	0.0579 (16)	
H5	0.5005	0.3793	0.6647	0.069*	
C6	0.4432 (4)	0.3534 (8)	0.8404 (7)	0.0593 (18)	
H6	0.4784	0.4038	0.8967	0.071*	
C7	0.6003 (4)	0.1950 (9)	0.4167 (9)	0.081 (2)	
H7A	0.6078	0.1492	0.3271	0.121*	
H7B	0.6472	0.2011	0.4637	0.121*	
H7C	0.5794	0.3047	0.4090	0.121*	
C8	0.4828 (3)	0.0677 (6)	0.4279 (7)	0.0504 (14)	
H8	0.4915	0.0421	0.3312	0.060*	
C9	0.4447 (3)	−0.0804 (7)	0.4942 (6)	0.0493 (14)	
C10	0.3836 (3)	−0.1693 (7)	0.4225 (7)	0.0465 (13)	
C11	0.3538 (3)	−0.3095 (7)	0.4869 (7)	0.0577 (16)	
H11	0.3719	−0.3431	0.5716	0.069*	
C12	0.2974 (3)	−0.3981 (7)	0.4246 (9)	0.0690 (18)	
H12	0.2784	−0.4924	0.4674	0.083*	
C13	0.2691 (4)	−0.3500 (9)	0.3017 (9)	0.073 (2)	
H13	0.2306	−0.4097	0.2613	0.087*	
C14	0.2984 (4)	−0.2114 (9)	0.2377 (8)	0.075 (2)	
H14	0.2794	−0.1774	0.1537	0.090*	
C15	0.3559 (3)	−0.1224 (8)	0.2978 (7)	0.0621 (17)	
H15	0.3758	−0.0303	0.2531	0.075*	

### Atomic displacement parameters (Å^2^)

**Table:**

	*U*^11^	*U*^22^	*U*^33^	*U*^12^	*U*^13^	*U*^23^
Cl1	0.1147 (15)	0.1100 (15)	0.0501 (11)	0.0357 (11)	0.0107 (12)	−0.0064 (10)
S1	0.0977 (11)	0.0519 (8)	0.0441 (8)	0.0100 (8)	−0.0060 (11)	0.0053 (8)
O1	0.072 (3)	0.068 (3)	0.045 (3)	−0.012 (2)	−0.002 (2)	0.001 (2)
O2	0.103 (3)	0.066 (3)	0.037 (3)	−0.010 (2)	−0.008 (3)	0.009 (2)
C1	0.074 (4)	0.054 (4)	0.042 (4)	0.022 (3)	0.005 (4)	−0.003 (3)
C2	0.075 (5)	0.061 (4)	0.071 (5)	0.002 (3)	0.014 (4)	−0.002 (4)
C3	0.073 (4)	0.054 (4)	0.066 (5)	0.000 (3)	−0.005 (4)	−0.006 (3)
C4	0.074 (4)	0.036 (3)	0.048 (4)	0.009 (3)	−0.003 (4)	0.001 (3)
C5	0.068 (4)	0.051 (4)	0.055 (4)	0.005 (3)	0.001 (4)	−0.004 (3)
C6	0.068 (4)	0.058 (4)	0.052 (4)	0.014 (3)	−0.010 (3)	−0.015 (3)
C7	0.087 (5)	0.092 (5)	0.063 (6)	−0.026 (4)	0.004 (5)	−0.004 (5)
C8	0.068 (3)	0.049 (3)	0.034 (3)	0.001 (3)	0.003 (3)	0.003 (3)
C9	0.075 (4)	0.046 (3)	0.028 (3)	0.009 (3)	0.009 (3)	0.002 (3)
C10	0.061 (3)	0.044 (3)	0.035 (3)	0.006 (3)	0.006 (3)	−0.002 (3)
C11	0.067 (4)	0.056 (4)	0.050 (4)	0.005 (3)	0.004 (3)	0.008 (3)
C12	0.073 (4)	0.053 (4)	0.081 (6)	−0.007 (3)	0.012 (5)	0.005 (4)
C13	0.073 (5)	0.076 (5)	0.069 (5)	−0.011 (3)	0.002 (4)	−0.011 (4)
C14	0.088 (5)	0.092 (5)	0.046 (5)	−0.003 (4)	−0.010 (4)	−0.006 (4)
C15	0.082 (4)	0.059 (4)	0.046 (4)	−0.013 (3)	−0.003 (4)	0.001 (3)

### Geometric parameters (Å, º)

**Table:**

Cl1—C1	1.741 (7)	C7—H7B	0.9600
S1—C4	1.780 (7)	C7—H7C	0.9600
S1—C8	1.853 (5)	C8—C9	1.517 (7)
O1—C8	1.386 (6)	C8—H8	0.9800
O1—C7	1.412 (8)	C9—C10	1.485 (8)
O2—C9	1.219 (7)	C10—C15	1.370 (9)
C1—C6	1.362 (9)	C10—C11	1.395 (8)
C1—C2	1.372 (10)	C11—C12	1.379 (9)
C2—C3	1.401 (10)	C11—H11	0.9300
C2—H2	0.9300	C12—C13	1.360 (11)
C3—C4	1.376 (9)	C12—H12	0.9300
C3—H3	0.9300	C13—C14	1.380 (9)
C4—C5	1.378 (8)	C13—H13	0.9300
C5—C6	1.381 (9)	C14—C15	1.387 (8)
C5—H5	0.9300	C14—H14	0.9300
C6—H6	0.9300	C15—H15	0.9300
C7—H7A	0.9600		
			
C4—S1—C8	101.5 (3)	O1—C8—S1	114.1 (4)
C8—O1—C7	114.1 (5)	C9—C8—S1	108.2 (4)
C6—C1—C2	121.6 (7)	O1—C8—H8	108.6
C6—C1—Cl1	119.7 (6)	C9—C8—H8	108.6
C2—C1—Cl1	118.6 (6)	S1—C8—H8	108.6
C1—C2—C3	118.2 (7)	O2—C9—C10	120.7 (5)
C1—C2—H2	120.9	O2—C9—C8	118.8 (5)
C3—C2—H2	120.9	C10—C9—C8	120.6 (5)
C4—C3—C2	120.6 (6)	C15—C10—C11	118.9 (6)
C4—C3—H3	119.7	C15—C10—C9	123.8 (5)
C2—C3—H3	119.7	C11—C10—C9	117.3 (5)
C3—C4—C5	119.6 (7)	C12—C11—C10	119.9 (7)
C3—C4—S1	119.7 (5)	C12—C11—H11	120.0
C5—C4—S1	120.6 (5)	C10—C11—H11	120.0
C4—C5—C6	120.0 (6)	C13—C12—C11	121.2 (6)
C4—C5—H5	120.0	C13—C12—H12	119.4
C6—C5—H5	120.0	C11—C12—H12	119.4
C1—C6—C5	119.9 (6)	C12—C13—C14	119.1 (7)
C1—C6—H6	120.1	C12—C13—H13	120.5
C5—C6—H6	120.1	C14—C13—H13	120.5
O1—C7—H7A	109.5	C13—C14—C15	120.5 (7)
O1—C7—H7B	109.5	C13—C14—H14	119.8
H7A—C7—H7B	109.5	C15—C14—H14	119.8
O1—C7—H7C	109.5	C10—C15—C14	120.4 (6)
H7A—C7—H7C	109.5	C10—C15—H15	119.8
H7B—C7—H7C	109.5	C14—C15—H15	119.8
O1—C8—C9	108.6 (5)		
			
C6—C1—C2—C3	0.8 (9)	O1—C8—C9—O2	19.3 (7)
Cl1—C1—C2—C3	179.3 (5)	S1—C8—C9—O2	−105.1 (5)
C1—C2—C3—C4	−1.2 (9)	O1—C8—C9—C10	−160.4 (5)
C2—C3—C4—C5	0.9 (9)	S1—C8—C9—C10	75.2 (6)
C2—C3—C4—S1	−177.2 (5)	O2—C9—C10—C15	177.7 (6)
C8—S1—C4—C3	−101.5 (5)	C8—C9—C10—C15	−2.5 (8)
C8—S1—C4—C5	80.5 (5)	O2—C9—C10—C11	−2.9 (8)
C3—C4—C5—C6	−0.2 (8)	C8—C9—C10—C11	176.9 (5)
S1—C4—C5—C6	177.9 (5)	C15—C10—C11—C12	0.0 (9)
C2—C1—C6—C5	0.0 (9)	C9—C10—C11—C12	−179.4 (5)
Cl1—C1—C6—C5	−178.5 (5)	C10—C11—C12—C13	−1.0 (9)
C4—C5—C6—C1	−0.3 (9)	C11—C12—C13—C14	1.0 (10)
C7—O1—C8—C9	163.9 (5)	C12—C13—C14—C15	0.1 (10)
C7—O1—C8—S1	−75.3 (6)	C11—C10—C15—C14	1.0 (10)
C4—S1—C8—O1	−63.7 (4)	C9—C10—C15—C14	−179.6 (5)
C4—S1—C8—C9	57.3 (5)	C13—C14—C15—C10	−1.1 (10)

### Hydrogen-bond geometry (Å, º)

**Table:**

*D*—H···*A*	*D*—H	H···*A*	*D*···*A*	*D*—H···*A*
C7—H7*A*···O2^i^	0.96	2.53	3.297 (9)	137
C8—H8···O2^i^	0.98	2.42	3.305 (8)	150

Symmetry code: (i) −*x*+1, −*y*, *z*−1/2.
